# Impact of diabetes mellitus type-2 on the outcomes following mitral transcatheter edge-to-edge repair (TEER): A meta-analysis

**DOI:** 10.1016/j.ahjo.2025.100574

**Published:** 2025-07-08

**Authors:** Himaja Dutt Chigurupati, Sivaram Neppala, Rahul Chikatimalla, Ayman Fath, Prakash Upreti, Jeffery Bolte, Muhammad Abdullah Naveed, Adishwar Rao, Osama Altaee, Yasar Sattar, Rupak Desai, Ralph A. DeFronzo, Jamal S. Rana, Timir K. Paul

**Affiliations:** a Department of Medicine, New York Medical College at Saint Michael's Medical Center, Newark, NJ, USA; b Department of Cardiology, University of Texas Health Sciences Center, San Antonio, TX, USA; c Department of Cardiology, Leonard M. Miller School of Medicine, University of Miami, Miami, FL, USA; d Sands-Constellation Heart Institute, Rochester Regional Health, Rochester, NY, USA; e Department of Medicine, Dow Medical College, Karachi, Pakistan; f Department of Internal Medicine, Guthrie Robert Packer Hospital, Sayre, PA, USA; g Department of Cardiology, West Virginia University Heart and Vascular Institute, Morgantown, WV, USA; h Independent Outcomes Researcher, Atlanta, GA, USA; i Division of Diabetes, University of Texas Health Sciences Center, San Antonio, TX, USA; j Department of Cardiology, The Permanente Medical Group, Oakland, CA, USA; k Department of Cardiovascular Sciences, Ascension St. Thomas Hospital/University of Tennessee Health Sciences Center, Nashville, TN, USA

**Keywords:** Diabetes mellitus, Mitral regurgitation, Mitral transcatheter edge-to-edge repair, Complications

## Abstract

**Background:**

Diabetes mellitus (DM) has been linked to unfavorable outcomes in patients undergoing Mitral Transcatheter Edge-to-Edge Repair (TEER). Nevertheless, the literature contains conflicting data. This meta-analysis aimed to assess the impact of DM on outcomes following Mitral TEER.

**Methods:**

We searched PubMed, Scopus, and Medline for studies reporting outcomes following mitral TEER in diabetic and non-diabetic patients. Using a random-effects model, we determined the pooled odds ratio (OR) for clinical outcomes in patients who underwent Mitral TEER, regardless of their diabetes status.

**Results:**

We included four studies with 2130 patients. DM was present in 31 % of the population, with a mean age of 73.9 (±8.2) years, 50.2 % of males, and 30 % of the population being obese. Patients with DM were more likely to be obese compared to patients without DM. In this meta-analysis, individuals with DM exhibited a higher 30-day MACCE (OR: 1.50, 95 % CI: 1.08–2.09, *p* = 0.02) and all-cause recurrent hospitalizations (OR: 1.36, 95 % CI: 1.07–1.72, *p* = 0.01) compared to those without diabetes. However, the difference in 30-day all-cause mortality (OR: 1.20, 95 % CI: 0.92–1.56, *p* = 0.19) and in-hospital all-cause mortality (OR: 0.92, 95 % CI: 0.51–1.67, *p* = 0.78) was not statistically significant between the two groups.

**Conclusion:**

DM is associated with an increased risk of 30-day MACCE and recurrent hospitalizations following Mitral TEER. Consequently, DM should be regarded as a predictor of adverse outcomes. Future, well-designed prospective randomized trials are necessary to evaluate the mid-term impact of DM on MACCE.

## Highlights

• Diabetes Mellitus (DM) is associated with increased risks of 30-day Major adverse cardiac and cerebrovascular events and recurrent hospitalizations in patients undergoing Mitral-TEER.
• The procedure duration was shorter among the individuals with diabetes than those without diabetes, and the number of implanted clips was comparable in both groups at 1.4 ± 0.6.
• No significant difference in in-hospital and 30-day mortality was noted between the groups
• We have not seen any significant difference in the mid-term mortality and MACCE between both the groups
• Patients with diabetes mellitus had a higher prevalence of comorbidities when compared to patients without diabetes and also higher surgical risk candidates.

## 1 Introduction

Mitral regurgitation (MR) is a common heart valve abnormality that affects approximately 2 % of the global population, and its prevalence increases with age [[1], [2], [3]]. Research indicates that moderate to severe MR can significantly heighten the risk of morbidity and mortality if left untreated, regardless of its cause or type [[4], [5], [6]]. While surgery has traditionally been the primary treatment for severe MR [7], nearly half of the patients are considered ineligible for surgery due to high risks [8].

In recent times, Mitral Transcatheter Edge-to-Edge Repair (TEER) has emerged as an alternative for patients who are not suitable for surgical intervention [9,12]. Studies such as the Endovascular Valve Edge-to-Edge Repair Study (EVEREST) and EVEREST II have demonstrated that the transcatheter approach is safer and more effective than medical treatment in reducing functional or degenerative MR [10,11] and lowering the need for repeat hospitalizations. It has also been associated with a 47 % and 38 % reduction in all-cause mortality at the 2-year follow-up [13]. However, despite its minimally invasive nature and positive outcomes, Mitral TEER has been linked to increased periprocedural complications necessitating hospitalization and higher mortality rates [[14], [15], [16]].

Diabetes mellitus (DM) is a well-established risk factor for cardiovascular diseases and is an independent predictor of morbidity and mortality in patients undergoing cardiac valve surgery and angioplasty [17,18]. It is closely related to mitral valve calcification [29,30], which is common in patients at high cardiovascular risk. Various factors such as pulmonary hypertension, heart failure, atrial fibrillation, and the severity and cause of MR have been identified as predictors of adverse clinical outcomes in patients undergoing Mitral TEER [[24], [25], [26], [27], [28]]. However, the impact of DM on the prognosis of Mitral TEER remains inconclusive due to conflicting findings from different studies [[19], [20], [21], [22], [23]]. The COAPT (Cardiovascular Outcomes Assessment of the Mitra Clip Percutaneous Therapy for Heart Failure Patients with Functional Mitral Regurgitation) trial revealed that diabetic patients undergoing Mitral TEER tended to have a higher rehospitalization rate for heart failure (HF) and higher all-cause mortality compared to those without diabetes [19,20]. Nevertheless, other studies indicated no significant impact of diabetes on adverse outcomes in patients with DM undergoing Mitral TEER [[21], [22], [23]].

Considering the contradictory results, a comprehensive meta-analysis was conducted to evaluate the effect of DM on outcomes, including all-cause mortality, major adverse cardiovascular and cerebrovascular events (MACCE), and recurrent hospitalizations in patients undergoing mitral valve repair.

## 2 Methods

### 2.1 Search and selection criteria

**Fig. 1:**
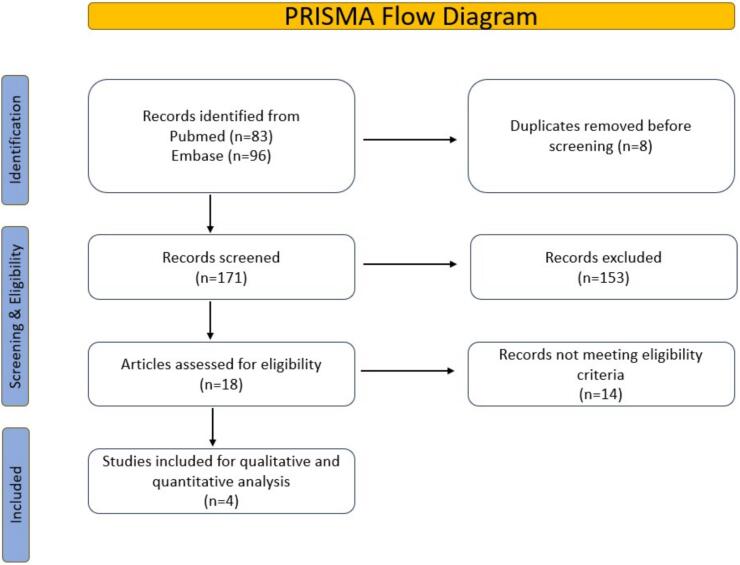
PRISMA flow diagram.

This systematic review was conducted according to the Cochrane Handbook of Systematic Reviews and followed the Preferred Reporting Items for Systematic Reviews and Meta-Analyses (PRISMA) [43] (Fig. 1 and Supplemental S-1) and AMSTAR-2 (Assessing the methodological quality of systematic reviews-2) guidelines checklist (Supplemental S-2) requirements. PubMed/Medline, Embase, and Scopus databases were systematically searched until January 2024 using specific keywords “Transcatheter edge-to-edge mitral valve repair,” “Transcatheter mitral valve repair,” “percutaneous mitral valve” OR “Transcutaneous mitral valve” combined with “Diabetes Mellitus,” “Diabetes Mellitus Type-2”, “DM-2”, and “DM.”. The search results were initially screened using the titles and abstracts, and potentially relevant studies were included for full-text review. The inclusion criteria consisted of prospective and retrospective human studies with a sample size of ≥20 subjects. In contrast, the exclusion criteria included studies with insufficient data, conference papers, review articles, editorials, case reports, studies with a sample size of <20, animal studies, and non-English-speaking studies. Duplicate studies were filtered, and the articles were transferred to Endnote library software. Two independent reviewers evaluated the remaining articles to ensure they met the established criteria, and the reference lists of the included studies were manually examined. The search strategy, research question, PICO, MeSH, and Keywords were mentioned in Supplemental S-3.

### 2.2 Study subjects

Following baseline characteristics were extracted from included studies: the number of subjects, age, gender, body mass index (BMI), and comorbidities, including hypertension, chronic obstructive pulmonary disease, acute kidney injury, atrial fibrillation, coronary artery disease, previous history of myocardial infarction, percutaneous coronary intervention, coronary artery bypass grafting, congestive heart failure, the New York Heart Association (NYHA) functional classification, and surgical risk scoring based on the Society of Thoracic Surgeons Predicted Risk of Mortality (STS-PROM) Reported echocardiographic parameters were also extracted, including left ventricular (LV) ejection fraction, left ventricular end-systolic and end-diastolic diameter, mitral regurgitation, and tricuspid regurgitation severity. Diabetes mellitus was defined under the criteria established in the respective included studies. This generally encompassed a prior diagnosis of diabetes mellitus (DM), administration of anti-diabetic medications, or the presence of biochemical markers such as HbA1c or fasting glucose levels.

### 2.3 Study outcomes

The primary outcomes examined were in-hospital all-cause mortality, MACCE (Major Adverse Cardiac and Cerebrovascular Events), recurrent heart failure hospitalizations, and 30-day all-cause mortality following Mitral TEER. We also assessed the mid-term results, specifically all-cause mortality and HF hospitalizations within the initial year. Secondary outcomes encompassed the quantity of implanted clips, procedural duration, and the reduction in mitral regurgitation during the periprocedural period. The definitions of major outcomes were provided in Supplemental Table 8.

### 2.4 Statistical analysis

We carefully documented the continuous variable, age, as mean ± standard deviation, and represented categorical variables as counts and percentages. The Shapiro-Wilk test was used to assess the normality of the continuous variables. A simple *t*-test was used to compare continuous variables, while a Chi-square test was employed for categorical variables. Odds ratios (OR) and their 95 % confidence intervals (CI) were calculated using multivariable logistic regression for the outcomes. The Joanna Briggs Institute (JBI) appraisal tool for meta-analyses of observational cohort or cross-sectional studies was utilized to evaluate the studies [31]. The regression analysis was adjusted for confounding factors, including age, sex, and relevant comorbidities such as diabetes, hypertension, congestive heart failure, renal disease, and obesity. Using funnel plots and Egger's test, the publication bias was evaluated for our study (Supplemental S-6). All reported *P*-values are two-sided, and the quality of the studies was assessed using the Newcastle-Ottawa Scale, with scores ranging from 0 to 8 [32]. This scale, frequently used in health sciences research, enabled us to evaluate study quality based on three main aspects: the selection of study groups, the comparability of the groups, and the ascertainment of exposure or outcome (Supplemental S-5). The analysis was performed using Open Meta [Analyst] software with a random-effects model for the pooled odds ratio. Forest plots were generated using STATA software version 18 (StataCorp, College Station, TX, USA).

## 3 Results

**Fig. 2:**
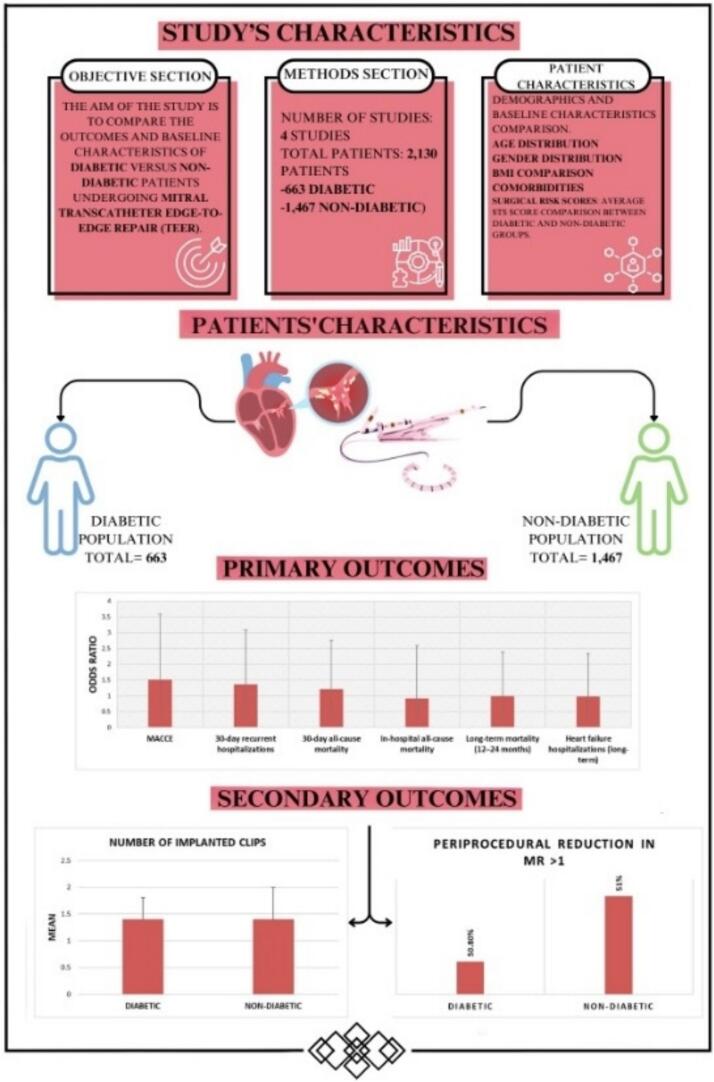
The central illustration presents a brief study on DM's impact on short-term TEER outcomes, including all-cause mortality, MACCE, and recurrent hospitalizations, along with characteristics and results.

We identified a total of 83 manuscripts from PubMed and 96 from Embase. After removing duplicates, animal studies, and abstracts, we screened 171 distinct records. According to our criteria, we ruled out 153 records. After applying our inclusion and exclusion criteria, 18 studies remained; however, we later excluded 11 of them. As a result, four studies with 2130 patients were incorporated into the final analysis following a quality assessment, as depicted in the PRISMA flowchart, with an average follow-up period of 30 (±15) days post-Mitral TEER (Fig. 1). The primary illustration in our manuscript clearly outlines the study design, patient demographics, and outcomes for patients undergoing mitral transcatheter edge-to-edge repair (TEER), contrasting those with diabetes mellitus against those without (Fig. 2).

### 3.1 Patient characteristics

The study included 2130 participants, an average age of 73.9 (±8.2) years, and a male proportion of 50.2 %. Individuals with diabetes mellitus (DM) were more likely to be overweight compared to those without it (average BMI of 28.1 ± 4.4 kg/m^2^ vs. 26.4 ± 5.1 kg/m^2^). They also faced a higher prevalence of hypertension, chronic obstructive pulmonary disease, renal disease, coronary artery disease, and prior myocardial infarction when compared to non-DM individuals; these differences did not reach statistical significance (*p* > 0.05) (see Table 1). Overall, those with DM exhibited a greater surgical risk than their non-DM counterparts, as demonstrated by mean logistic Euro SCORE and STS-PROM values of 17.3 and 6.35, respectively, compared to 16 and 5.1 for non-DM individuals. While patients with DM showed numerically elevated STS scores, EuroSCORE, and left ventricular end-diastolic diameter (LVEDD) compared to non-diabetic patients, these differences did not reach statistical significance. Furthermore, the ejection fraction was lower in the DM group (36.7 % vs. 39 %), and they had a larger left ventricular end-diastolic diameter (60.7 mm vs. 59.9 mm) than those without DM. The average number of clips implanted was similar in both groups, at 1.4 ± 0.6. The clip implantation procedure duration was shorter for the DM group compared to non-DM patients (114.99 ± 52.39 vs. 117 ± 67.71). The procedural duration for diabetic patients was comparable to that of non-diabetic patients, as noted in the included studies. Study characteristics are mentioned in Fig. 2. Additionally, the primary endpoint analysis's symmetrical funnel plots further validate the robustness of our findings (Supplemental S6).

**Table 1:** Baseline characteristics of included studies with and without diabetes undergoing mitral TEER:

Diabetic population						Non-diabetic population						*P* value
Author	Bahira Sahim	Michael Paukovitsch	Annemarie Kirschfink	Katharina Hellhammer	Subtotal	Bahira Sahim	Michael Paukovitsch	Annemarie Kirschfink	Katharina Hellhammer	Subtotal	Totals	
Year	2021	2023	2022	2014		2021	2023	2022	2014			
Mean Age	71 ± 10.1	76 ± 8.3	77	68 ± 9.3	74.2	72.9 ± 11.7	77.6 ± 9.1	79	73 ± 11.3	76.5	75.8	0.358
Total Number	229	306	109	19	663	385	812	231	39	1467	2130	
Male No. (%)	62 (27 %)	182 (59 %)	73 (67 %)	16 (84 %)	333 (50 %)	65 (17 %)	468 (58 %)	145 (63 %)	23 (59 %)	701 (48 %)	1034 (49 %)	0.558
Female No. (%)	167 (73 %)	124 (41 %)	36 (33 %)	3 (16 %)	330 (50 %)	320 (83 %)	344 (42 %)	86 (37 %)	16 (41 %)	766 (52 %)	1096 (48 %)	
BMI	28.6	27.7	N/A	28	28.1	26.1	25.5	N/A	26	25.7	26.4	0.002
HTN No. (%)	89 (39 %)	269 (89 %)	97 (89 %)	19 (100 %)	474 (71 %)	75 (19 %)	624 (77 %)	172 (74 %)	36 (92 %)	907 (62 %)	1381 (65 %)	0.544
COPD No. (%)	27 (12 %)	40 (13 %)	N/A	5 (26 %)	72 (13 %)	21 (5 %)	82 (10 %)	N/A	9 (23 %)	112 (9 %)	184 (10 %)	0.57
AKI/Renal Disease No. (%)	65 (28 %)	241 (79 %)	84 (77 %)	11 (58 %)	401 (60 %)	52 (14 %)	583 (72 %)	160 (69 %)	21 (54 %)	816 (56 %)	1217 (57 %)	0.66
Atrial Fibrillation No. (%)	53 (23 %)	186 (61 %)	74 (68 %)	5 (26 %)	318 (48 %)	56 (15 %)	542 (67 %)	166 (72 %)	15 (38 %)	779 (53 %)	1097 (52 %)	0.85
CAD No. (%)	79 (34 %)	230 (75 %)	N/A	11 (58 %)	320 (58 %)	69 (18 %)	509 (63 %)	N/A	24 (62 %)	602 (49 %)	922 (52 %)	0.696
Previous MI, No. (%)	55 (24 %)	108 (35 %)	99 (91 %)	9 (47 %)	271 (41 %)	49 (13 %)	167 (21 %)	154 (67 %)	13 (33 %)	383 (26 %)	654 (31 %)	0.437
Prior CABG No. (%)	42 (18 %)	N/A	38 (35 %)	5 (26 %)	85 (24 %)	39 (10 %)	N/A	45 (19 %)	11 (28 %)	95 (15 %)	180 (18 %)	0.363
Prior PCI No. (%)	50 (22 %)	N/A	61 (56 %)	1 (5 %)	112 (31 %)	44 (11 %)	N/A	109 (47 %)	1 (3 %)	154 (24 %)	266 (26 %)	0.735
NYHA I No. (%)	35 (15 %)	39 (13 %)	16 (15 %)	N/A	90 (14 %)	41 (11 %)	126 (16 %)	25 (11 %)	N/A	192 (13 %)	282 (14 %)	0.422
NYHA II No. (%)	86 (38 %)	175 (57 %)	58 (53 %)	5 (26 %)	324 (49 %)	81 (21 %)	484 (60 %)	155 (67 %)	20 (51 %)	740 (50 %)	1064 (50 %)	0.635
NYHA III No. (%)	43 (19 %)	92 (30 %)	35 (32 %)	4 (21 %)	174 (26 %)	36 (9 %)	202 (25 %)	49 (21 %)	15 (38 %)	302 (21 %)	476 (22 %)	0.754
LVEF (%)	32.1	40.7	37	37	36.7	30.9	44.4	40	45	40.1	39	0.404
LVEDD (mm)	61	61	59	61 14	60.7	62	59.3 ± 11.6	56	59 ± 11	59.5	59.9	0.343
LVESD (mm)	52	47	48	N/A	48.9	53	44.4 ± 13.8	44	N/A	46.7	47.4	0.612
Mod-Severe MR No. (%)	100 (44 %)	228 (75 %)	41 (38 %)	18 (95 %)	387 (58 %)	100 (26 %)	385 (47 %)	102 (44 %)	30 (77 %)	617 (42 %)	1004 (47 %)	0.428
Mod-Severe TR No. (%)	1 (0 %)	83 (27 %)	59 (54 %)	N/A	143 (22 %)	N/A	258 (32 %)	115 (50 %)	N/A	373 (26 %)	516 (25 %)	0.988
Average STS Score	6.9	5.8	N/A	N/A	6.3	5.1	5.2	N/A	N/A	5.2	5.5	0.275
EURO Score	N/A	9.1	24	18.7	17.2	N/A	7.9	23	18.6	16.5	16.8	0.908
Procedure time	158.5 ± 73.5	90.24 ± 51.76	90.25 ± 41.3	121 ± 43	115	168.6 ± 127.3	86.3 ± 55.1	93.52 ± 51.3	120 ± 37	117	116.05	0.935

Concerning the etiology of MR, one study (Paukovitsch et al.) presented subgroup data for both functional and degenerative MR, indicating significantly poorer outcomes in diabetic patients with degenerative MR. Another study (Shahim et al.) concentrated solely on functional MR, whereas the remaining studies encompassed mixed or unspecified populations. Due to the inconsistency in stratification across the included studies, conducting a subgroup meta-analysis based on the etiology of MR was unfeasible. Nonetheless, this heterogeneity in etiology underscores the need for further research into the differential outcomes associated with various MR origin subtypes (Table 2). Data regarding the reduction of post-procedural MR were available in three of the four included studies. While all three documented a significant improvement in MR following TEER, none of them provided subgroup analyses based on diabetes status. One study (Shahim et al.) observed a reduction of ≥2 grades in MR in 95 % of patients, and another (Hellhammer et al.) reported a decrease to mild or lesser severity in most cases. Due to inconsistent reporting and the absence of stratification, MR reduction was not incorporated into the pooled regression analysis (Table 3).

**Table 2:** Mitral regurgitation etiology.

Study	MR etiology
Paukovitsch et al.	Reported separately as Degenerative and Functional MR
Shahim et al. (COAPT)	Functional MR only
Kirschfink et al.	Primarily Functional MR
Hellhammer et al.	Mixed etiology (both FMR and DMR included, not stratified)

**Table 3:** Post-procedural MR reduction.

Study	Post-procedural MR reduction
Paukovitsch et al.	Reported: ≥1-grade MR reduction in most patients; not stratified by DM
Shahim et al. (COAPT)	Reported: ≥2-grade MR reduction in 95 % overall; DM-specific data not provided
Kirschfink et al.	Not reported
Hellhammer et al.	Reported: MR reduced to mild or less in most; not stratified by DM

MR = Mitral Regurgitation. MR >1 refers to post-procedural MR reduction greater than one grade, indicating significant improvement.

### 3.2 Primary outcomes

**Fig. 3:**
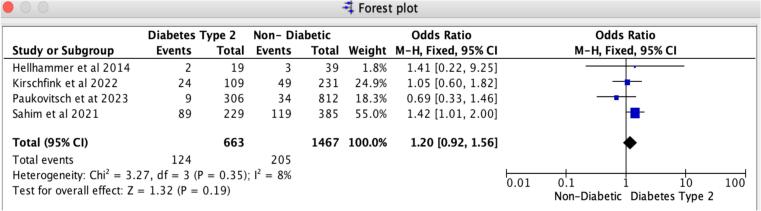
Forrest Plot for 30-day all-cause mortality. Forrest plot predicting 30-day all-cause mortality in patients undergoing Mitral TEER with Diabetes mellitus type-2 vs. without Diabetes mellitus. CI: confidence interval, OR: odds ratio, TEER: transcatheter edge-to-edge repair.

**Fig. 4:**
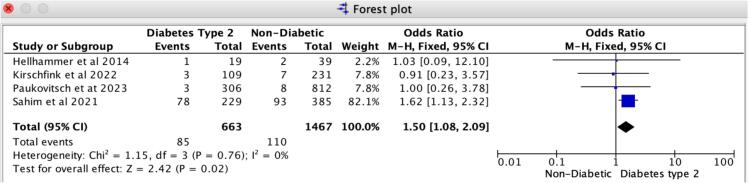
Forrest plot for 30-day MACCE. Forrest plot predicting 30-day MACCE in patients undergoing Mitral TEER with Diabetes mellitus type-2 vs. without Diabetes mellitus. CI: confidence interval, MACCE: Major adverse Cardiac and Cerebrovascular Events. OR: odds ratio, TEER: transcatheter edge-to-edge repair.

**Fig. 5:**
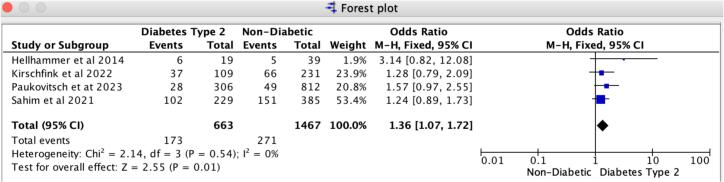
Forrest plot for 30-day all-cause recurrent hospitalizations. Forrest plot predicting 30-day all-cause recurrent hospitalizations in patients undergoing Mitral TEER with Diabetes mellitus type-2 vs. without Diabetes mellitus. CI: confidence interval, OR: odds ratio, TEER: transcatheter edge-to-edge repair.

**Fig. 6:**
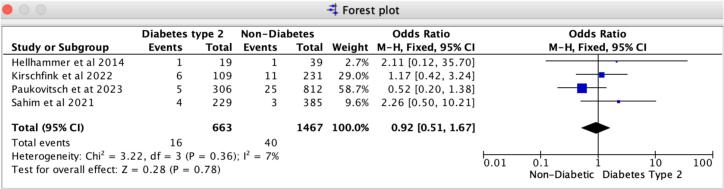
Forrest plot for in-hospital mortality. Forrest plot predicting in-hospital all-cause mortality in patients undergoing Mitral TEER with Diabetes mellitus type-2 vs. without Diabetes mellitus. CI: confidence interval, OR: odds ratio, TEER: transcatheter edge-to-edge repair.

In patients receiving Mitral TEER, diabetes is associated with a notably heightened risk of 30-day MACCE and recurrent hospitalizations compared to those without diabetes. Our findings reveal that the odds ratio for 30-day major adverse cardiovascular and cerebrovascular events (MACCE) is 1.50 (95 % CI: 1.08–2.09, *p* = 0.02), reflecting a strong association with no evidence of heterogeneity (I2 = 0 %). For 30-day all-cause recurrent hospitalizations, the odds ratio is 1.36 (95 % CI: 1.07–1.72, *p* = 0.01), with no heterogeneity observed (I2 = 0 %) (Fig. 4, Fig. 5). Likewise, the leave-one-out sensitivity analysis for 30-day all-cause recurrent hospitalizations revealed no significant differences when excluding any single study, as illustrated in Supplemental S7. However, for the 30-day MACCE, we noted a variation in the odds ratio when excluding Sahim et al. 2021, while the odds ratio remained consistent across the other studies, as shown in Supplemental S7. Additionally, we found no notable differences in in-hospital and 30-day all-cause mortality rates between patients with diabetes and those without. Specifically, the odds ratio for 30-day all-cause mortality is 1.20 (95 % CI: 0.92–1.56, *p* = 0.19), revealing slight heterogeneity (I2 = 8 %); likewise, the odds ratio for in-hospital all-cause mortality is 0.92 (95 % CI: 0.51–1.67, *p* = 0.78), with slight heterogeneity (I2 = 7 %) (Fig. 3, Fig. 6).

While the average follow-up duration in various studies was 30 days, some specifically reported outcomes at 1-year intervals. These studies were separately analyzed to assess mid-term results. Moreover, mid-term mortality (12–24 months) was examined in two extensive studies with 954 patients who underwent mitral TEER. The findings showed no significant differences in all-cause mortality (OR: 0.99, 95 % CI: 0.69–1.41, *p* = 0.96) or heart failure hospitalization rates (OR: 0.98, 95 % CI: 0.70–1.36, *p* = 0.90) between patients with DM and those without it.

### 3.3 Secondary outcomes

The study revealed that the number of implanted clips (1.4 ± 0.4 vs. 1.4 ± 0.6), procedural duration for mitral TEER (114.99 ± 52.39 vs. 117 ± 67.7), and the periprocedural decrease in mitral regurgitation >1 (50.8 % vs. 51 %) were statistically comparable between diabetic individuals and non-diabetic individuals.

## 4 Discussion

This is the first meta-analysis investigating the impact of pre-procedural diabetes mellitus (DM) on short-term outcomes after Mitral Transcatheter Edge-to-Edge Repair (TEER). It includes four studies and 2130 patients. The results indicate that pre-procedural DM is associated with increased risks of recurrent hospitalizations and major adverse cardiovascular and cerebrovascular events (MACCE). Still, it shows no significant difference in 30-day all-cause mortality and in-hospital mortality compared to patients without diabetes. Furthermore, while patients with DM exhibited more notable occurrences of comorbidities, such as CAD, CKD, and HF, these variations did not achieve statistical significance (*p* > 0.05). This implies a numerical trend rather than a statistically confirmed association. This finding suggests a numerical trend rather than a confirmed statistical association. Meanwhile, the two groups did not significantly differ in the number of clips implanted or the surgical/procedural time.

Numerous studies have established that individuals with DM often exhibit an unfavorable cardiovascular risk profile due to the gradual impact of DM on myocardial structure and function over time [[33], [34], [35], [36]]. Furthermore, research suggests a link between DM and the worsening of left ventricular remodeling in patients with MR [37]. Prior studies have emphasized that diabetic patients undergoing surgical mitral valve repair face elevated risks of both early and late mortality [[37], [38], [39], [40]]. They are more susceptible to recurrent hospitalizations due to HF [41].

Mitral TEER is a less invasive alternative to surgical mitral valve replacement and is recognized as safe and effective for high-risk patient populations [9,11,42]. Several studies have investigated the impact of DM on the prognosis of Mitral TEER, yielding conflicting results. Our analysis showed that patients with DM faced higher risks of recurrent hospitalizations after Mitral TEER. These results align with those of Shahim et al., who also discovered that diabetic patients have an increased rate of hospitalization for HF [19]. They further recommended that transcatheter mitral valve repair could be a more favorable option for diabetic patients compared to guideline-directed medical therapy (GDMT) alone.

On the other hand, a more recent study including 1118 patients also indicated an increased likelihood of HF hospitalizations in patients with diabetes, affirming the present study's findings [23]. Furthermore, these studies also demonstrated that diabetic patients who underwent Mitral TEER had higher chances of experiencing all-cause mortality and MACE [20,34]. Diabetes significantly impacts outcomes in coronary artery and valvular diseases, but its role in M-TEER differs. Unlike surgical procedures, where calcification plays a significant role, diabetes in M-TEER may lead to worse outcomes due to associated comorbidities like CAD, HFrEF, prior myocardial infarction, and CKD [23,44]. Another significant factor influencing early outcomes following TEER is the degree of MR reduction attained during the procedure. Multiple studies have demonstrated that a greater reduction in MR is independently correlated with decreased mortality and reduced hospitalization rates following Mitra Clip implantation [24,25]. Although our analysis incorporated data on MR reduction (specifically, MR greater than one grade), inconsistencies in reporting across studies prevented its inclusion in regression models. In our pooled analysis, the reduction in MR post-procedure was numerically comparable between diabetic and non-diabetic groups. Nevertheless, this variable may continue to serve a mediating role and warrants further investigation, particularly considering the structural and functional remodeling of the mitral apparatus associated with diabetes. These factors contribute to poorer post-procedural outcomes, explaining the observed diabetes-related risk trends in this patient group.

Nevertheless, our study did not find a significant difference in in-hospital all-cause mortality between the two groups, possibly due to the smaller number of Mitral TEER patients in our meta-analysis. The COAPT study illustrated that mortality and heart failure hospitalizations were lower in patients who received Mitral TEER plus GDMT compared to those who received GDMT alone, both in patients with diabetes (51.4 % vs. 70.4 %) and those without diabetes (41.3 % vs. 64.8 %). Mitral TEER plus GDMT consistently reduced 2-year all-cause mortality and heart failure hospitalizations in patients with and without diabetes in comparison to GDMT alone [19]. Conversely, the study by Kirschfink et al. on 340 patients who underwent Mitral TEER with moderate to severe MR found no significant difference in all-cause mortality or hospitalizations between diabetic and non-diabetic patients [21]. Similarly, Hellmmer et al.'s study on 58 patients found no significant variance in morbidity and mortality among diabetic and non-diabetic patients who underwent Mitral TEER. Our study has shown that stronger associations were observed at 30 days compared to mid-term, which may be partly attributable to the fact that fewer studies were conducted with extended follow-up beyond two years. This limitation could result in diminished statistical power in the mid-term analysis. The other reason may be attributable to the acute metabolic and inflammatory consequences, including endothelial dysfunction, increased thrombogenicity, and heightened inflammatory responses following the procedure. These mechanisms may contribute to early complications such as thrombotic events, hemodynamic instability, and procedural failure. Over time, however, the effect of diabetes may be attenuated by improved post-procedural management, evolving pharmacologic therapies, and careful patient selection [45]. Additionally, diabetes-related outcomes may differ based on MR etiology, with secondary MR patients potentially having a different risk profile due to a higher prevalence of concomitant heart failure and coronary artery disease [46].

Furthermore, the underlying etiology of MR—whether functional or degenerative—may influence the relationship between diabetes and post-TEER outcomes. In particular, Paukovitsch et al. found that diabetics with degenerative MR had significantly worse outcomes compared to non-diabetics, whereas this pattern was not observed in those with functional MR [15]. These findings suggest that MR etiology may act as an effect modifier, potentially amplifying the adverse impact of diabetes in the degenerative subgroup. While our meta-analysis included studies with mixed MR populations, most did not stratify outcomes based on etiology, which limited our ability to conduct a subgroup analysis. Future studies should investigate the interaction between MR etiology and glycemic status to guide more personalized risk assessment.

Elevated glycated hemoglobin (HbA1c) levels are strongly associated with a heightened risk of complications following cardiovascular procedures. Research has consistently shown that inadequate glycemic control increases the likelihood of complications after percutaneous coronary interventions (PCI) in patients with DM and that enhanced glycemic control can lead to significantly improved outcomes [47,48]. In a similar vein, studies reveal that mitral TEER is equally safe and effective for patients with severe MR and DM compared to their non-diabetic counterparts; however, this population still faces an increased risk of adverse outcomes [21,22]. Given the well-established link between hyperglycemia and detrimental cardiovascular events, there is an urgent need for further exploration into the prognostic implications of HbA1c levels in patients undergoing mitral TEER. Such research could illuminate critical pathways to optimize care and improve patient outcomes in this vulnerable group.

It's important to note that most existing literature focuses on short-term outcomes after Mitral TEER, with only a few studies reporting mid-term outcomes over 24 months [19,21]. These mid-term studies revealed no significant difference in all-cause mortality and HF hospitalizations. Diabetes was not correlated with mid-term mortality following Mitral TEER, but the scarcity of studies warrants caution in drawing this conclusion. The insufficient data on medical treatments, particularly for modern agents such as SGLT2 inhibitors, represents a significant constraint. Given their proven advantages in treating heart failure and diabetes, the increasing use of these agents could have improved mid-term outcomes, possibly lessening the harmful impacts of diabetes in this context. Further mid-term randomized studies are necessary to assess the impact of diabetes on outcomes after Mitral TEER.

## 5 Limitations

This meta-analysis presents certain limitations that should be acknowledged. These include the potential for selection bias stemming from the inclusion of single-center studies and the absence of randomization, which might have led to lingering confounding factors. Moreover, several studies considered in the analysis did not provide essential baseline data, such as accurate information on heart catheterizations, medication usage, and laboratory results—all of which could have influenced the overall outcomes of the studies. Additionally, the small sample sizes and relatively short follow-up periods following TEER have limited the analysis of some studies. Furthermore, we were unable to conduct stratified quantitative analysis based on MR etiology due to inconsistent reporting across the included studies. Additionally, the lack of uniform reporting of post-procedural MR reduction across studies limited its inclusion in regression analysis.

This meta-analysis utilizes study-level data instead of patient-level data, which restricts the capacity for individual patient adjustments and may introduce potential confounders that were not accounted for. Furthermore, since only four studies are included in this analysis, there exists a significant possibility of selection bias, which should be considered during interpretation.

It is essential to note that a single study can have a substantial impact on the results. However, we have mitigated this concern by performing a comprehensive evaluation, which included a sensitivity analysis and an examination of funnel plots. Given the observational nature of this study, it is essential to emphasize the need for extensive, randomized, prospective studies with more extended follow-up periods.

## 6 Conclusion

The results of this meta-analysis powerfully illuminate a significant association between diabetes mellitus (DM) and an increased risk of repeat hospitalizations, as well as major adverse cardiovascular and cerebrovascular events (MACCE) following mitral transcatheter edge-to-edge repair (TEER). Thus, DM should be viewed as a medical condition critical predictor of adverse outcomes in this context. Furthermore, optimizing glycated hemoglobin levels before the procedure can transform outcomes for this patient population. While our analysis found no clinically meaningful differences in in-hospital and 30-day mortality rates between the two groups, it is vital to recognize that most of the analyzed data concentrated on short-term follow-ups following mitral TEER. Consequently, there is an urgent need for prospective studies to meticulously examine the mid-term effects of DM on mortality and MACCE in patients undergoing mitral TEER, thereby enriching our comprehension of this essential relationship.

## CRediT authorship contribution statement

**Himaja Dutt Chigurupati:** Writing – original draft, Validation. **Sivaram Neppala:** Writing – review & editing, Writing – original draft, Supervision, Methodology, Conceptualization. **Rahul Chikatimalla:** Methodology, Formal analysis. **Ayman Fath:** Writing – review & editing. **Prakash Upreti:** Methodology, Data curation. **Jeffery Bolte:** Visualization. **Muhammad Abdullah Naveed:** Visualization, Validation. **Adishwar Rao:** Visualization, Validation, Methodology. **Osama Altaee:** Writing – review & editing, Supervision. **Yasar Sattar:** Writing – review & editing, Supervision. **Rupak Desai:** Writing – review & editing, Supervision. **Ralph A. DeFronzo:** Writing – review & editing, Supervision, Resources, Project administration. **Jamal S. Rana:** Writing – review & editing, Supervision, Resources. **Timir K. Paul:** Writing – review & editing, Supervision, Project administration.

## Ethics approval and consent to participate

Not applicable.

## Funding

The authors received no extramural funding for the study.

## Declaration of competing interest

All authors have no conflict of interest to disclose that is related to this article.

## Appendix A Supplementary data

Supplementary material Image 1
