# Supplementary material for: Impact of diabetes mellitus type-2 on the outcomes following mitral transcatheter edge-to-edge repair (TEER): A meta-analysis
Source: Am Heart J Plus. 2025 Jul 8;57:100574. doi: 10.1016/j.ahjo.2025.100574 (PMC12281527; doi:10.1016/j.ahjo.2025.100574)
Supplement: Supplementary file 1 — Supplementary material [file mmc1.docx]

**Supplemental Files Index**

1. Supplemental S1: Preferred Reporting Items for Systematic Reviews and Meta-analyses (PRISMA) checklist
2. Supplemental S2: AMSTAR-2 (Assessing the methodological quality of systematic reviews-2) Guidelines checklist
3. Supplemental S3: Research Question, PICO, MeSH, Keywords and Search Strategy
4. Supplemental S4: Risk of bias (ROB) assessment for randomized control trials
5. Supplemental S5: Newcastle-Ottawa Scale for assessment of bias for observational studies
6. Supplemental S6: Egger’s test and funnel plots to check for publication bias
7. Supplemental S7: Leave one-out sensitivity testing
8. Supplemental S8: Definitions of major outcomes

**Supplemental S1: Preferred Reporting Items for Systematic Reviews and Meta-analyses (PRISMA) checklist**


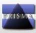
**PRISMA Checklist**

| **Section and**  **Topic** | **Item**  **#** | **Checklist item** | **Location**  **where item is reported** |
| --- | --- | --- | --- |
| **TITLE** | | |  |
| Title | 1 | Identify the report as a systematic review. | 1 |
| **ABSTRACT** | | |  |
| Abstract | 2 | See the PRISMA 2020 for Abstracts checklist. | 2 |
| **INTRODUCTION** | | |  |
| Rationale | 3 | Describe the rationale for the review in the context of existing knowledge. | 3 |
| Objectives | 4 | Provide an explicit statement of the objective(s) or question(s) the review addresses. | 3 |
| **METHODS** | | |  |
| Eligibility criteria | 5 | Specify the inclusion and exclusion criteria for the review and how studies were grouped for the syntheses. | 4 |
| Information  sources | 6 | Specify all databases, registers, websites, organizations, reference lists and other sources searched or consulted to identify studies. Specify the date when each source was last searched or consulted. | 4 |
| Search strategy | 7 | Present the full search strategies for all databases, registers and websites, including any filters and limits used. | 4 |
| Selection process | 8 | Specify the methods used to decide whether a study met the inclusion criteria of the review, including how many reviewers screened each record and each report retrieved, whether they worked independently, and if applicable, details of automation tools used in the process. | 4 |
| Data collection  process | 9 | Specify the methods used to collect data from reports, including how many reviewers collected data from each report, whether they worked independently, any processes for obtaining or confirming data from study investigators, and if applicable, details of automation tools used in the process. | 4 |
| Data items | 10a | List and define all outcomes for which data were sought. Specify whether all results that were compatible with each outcome domain in each study were sought (e.g. for all measures, time points, analyses), and if not, the methods used to decide which results to collect. | 4 |
|  | 10b | List and define all other variables for which data were sought (e.g. participant and intervention characteristics, funding sources). Describe any assumptions made about any missing or unclear information. | 5 |
| Study risk of bias assessment | 11 | Specify the methods used to assess risk of bias in the included studies, including details of the tool(s) used, how many reviewers assessed each study and whether they worked independently, and if applicable, details of automation tools used in the process. | 5 |
| Effect measures | 12 | Specify for each outcome the effect measure(s) (e.g. risk ratio, mean difference) used in the synthesis or presentation of results. | 5 |
| Synthesis  methods | 13a | Describe the processes used to decide which studies were eligible for each synthesis (e.g. tabulating the study intervention characteristics and comparing against the planned groups for each synthesis (item #5)). | 5 |
|  | 13b | Describe any methods required to prepare the data for presentation or synthesis, such as handling of missing summary statistics, or data conversions. | 5 |
|  | 13c | Describe any methods used to tabulate or visually display results of individual studies and syntheses. | 5 |
|  | 13d | Describe any methods used to synthesize results and provide a rationale for the choice(s). If meta-analysis was performed, describe the model(s), method(s) to identify the presence and extent of statistical heterogeneity, and software package(s) used. | 5 |
|  | 13e | Describe any methods used to explore possible causes of heterogeneity among study results (e.g. subgroup analysis, meta-regression). | 5 |
|  | 13f | Describe any sensitivity analyses conducted to assess robustness of the synthesized results. | 5 |
| Reporting bias  assessment | 14 | Describe any methods used to assess risk of bias due to missing results in a synthesis (arising from reporting biases). | 5 |
| Certainty  assessment | 15 | Describe any methods used to assess certainty (or confidence) in the body of evidence for an outcome. | 5 |


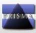
**PRISMA 2020 Checklist**

| **Section and**  **Topic** | **Item**  **#** | **Checklist item** | **Location**  **where item  is reported** |
| --- | --- | --- | --- |
| **RESULTS** | | |  |
| Study selection | 16a | Describe the results of the search and selection process, from the number of records identified in the search to the number of studies included in the review, ideally using a flow diagram. | 4 |
|  | 16b | Cite studies that might appear to meet the inclusion criteria, but which were excluded, and explain why they were excluded. | 4 |
| Study  characteristics | 17 | Cite each included study and present its characteristics. | Table 1 |
| Risk of bias in  studies | 18 | Present assessments of risk of bias for each included study. | Supplemental file |
| Results of  individual studies | 19 | For all outcomes, present, for each study: (a) summary statistics for each group (where appropriate) and (b) an effect estimates and its precision (e.g. confidence/credible interval), ideally using structured tables or plots. |  |
| Results of  syntheses | 20a | For each synthesis, briefly summarize the characteristics and risk of bias among contributing studies. | - |
|  | 20b | Present results of all statistical syntheses conducted. If meta-analysis was done, present for each the summary estimate and its precision (e.g.  confidence/credible interval) and measures of statistical heterogeneity. If comparing groups, describe the direction of the effect. | - |
|  | 20c | Present results of all investigations of possible causes of heterogeneity among study results. | - |
|  | 20d | Present results of all sensitivity analyses conducted to assess the robustness of the synthesized results. | - |
| Reporting biases | 21 | Present assessments of risk of bias due to missing results (arising from reporting biases) for each synthesis assessed. | - |
| Certainty of  evidence | 22 | Present assessments of certainty (or confidence) in the body of evidence for each outcome assessed. | - |
| **DISCUSSION** | | |  |
| Discussion | 23a | Provide a general interpretation of the results in the context of other evidence. | 6-7 |
|  | 23b | Discuss any limitations of the evidence included in the review. | 7 |
|  | 23c | Discuss any limitations of the review processes used. | 7 |
|  | 23d | Discuss implications of the results for practice, policy, and future research. | 7 |
| **OTHER INFORMATION** | | |  |
| Registration and protocol | 24a | Provide registration information for the review, including register name and registration number, or state that the review was not registered. | - |
|  | 24b | Indicate where the review protocol can be accessed, or state that a protocol was not prepared. | - |
|  | 24c | Describe and explain any amendments to information provided at registration or in the protocol. | - |
| Support | 25 | Describe sources of financial or non-financial support for the review, and the role of the funders or sponsors in the review. | - |
| Competing  interests | 26 | Declare any competing interests of review authors. | - |
| Availability of  data, code and  other materials | 27 | Report which of the following are publicly available and where they can be found: template data collection forms; data extracted from included studies; data used for all analyses; analytic code; any other materials used in the review. | - |

*From:* Page MJ, McKenzie JE, Bossuyt PM, Boutron I, Hoffmann TC, Mulrow CD, et al. The PRISMA 2020 statement: an updated guideline for reporting systematic reviews. BMJ 2021;372:n71. doi:  10.1136/bmj.n71

For more information, visit: <http://www.prisma-statement.org/>

**Supplemental S2: AMSTAR-2 (Assessing the methodological quality of systematic reviews-2) Guidelines checklist**

**
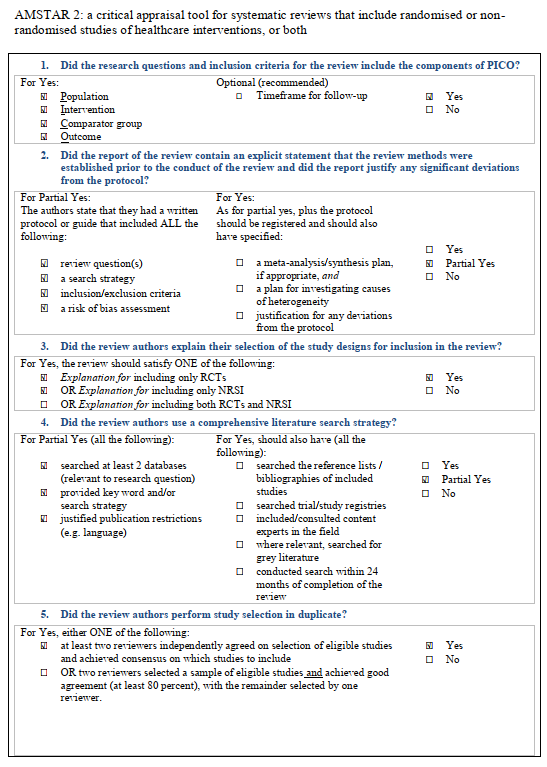
**

**
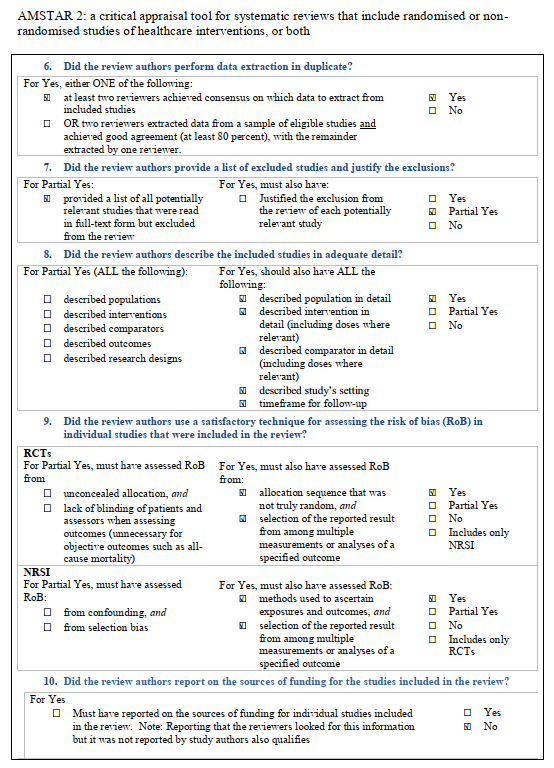
**

**
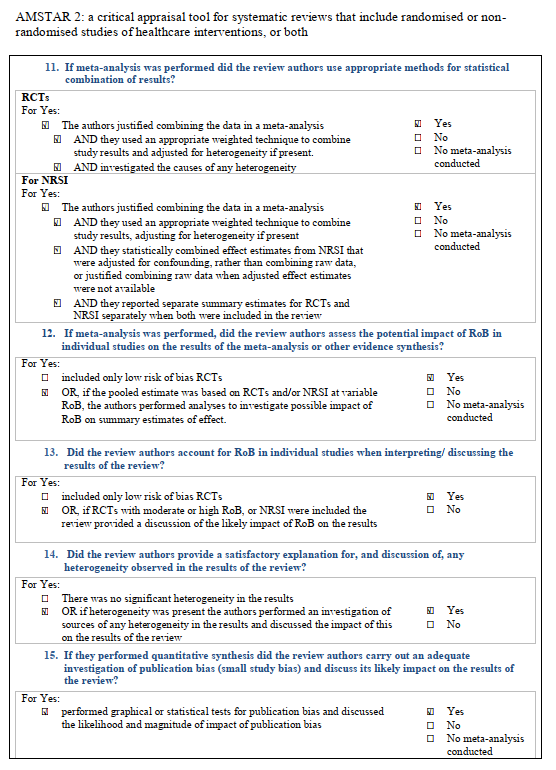
**

**
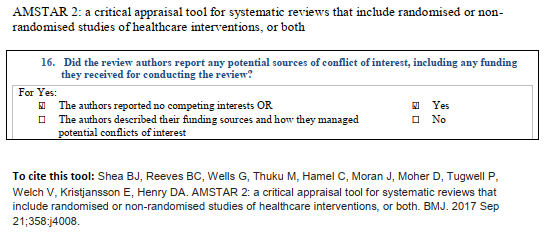
**

**Supplemental S3: Research Question, PICO, MeSH, Keywords, and Search Strategy**

**Research Question:** *The impact of Diabetes Mellitus on short-term outcomes after Mitral Transcatheter Edge-to-Edge Repair (TEER)*

**PICO:**

**Population:** Patients undergoing TEER of mitral valve

**Intervention:** Patients with diabetes mellitus

**Comparison:** Patients without diabetes mellitus

**Outcome:**

1) **Primary outcome 30-day** *all‐cause mortality, major adverse cardiac and cerebrovascular events (MACCE), recurrent hospitalizations, in-hospital mortality*

**2) Secondary outcomes** *included procedure time, number of clips, and reduction in mitral regurgitation.*

**Study type:** Odds ratio to compare binary outcomes and standard mean difference to compare continuous outcomes meta-analyses.

**MeSH Terms & Keywords:**

*Diabetes mellitus*

*DM*

*Mitral Valve [Mesh]*

*Transcatheter edge-to-edge mitral valve repair*

*Transcatheter mitral valve repair*

*Percutaneous mitral valve*

*Transcutaneous mitral valve*
**Detailed search strategy for each of the included databases.**

| **Database** | **Search Strategy** | **Articles retrieved** |
| --- | --- | --- |
| PubMed | (("Diabetes"[Mesh] OR "Diabetes type-2" OR "DM"[Title/Abstract] OR "DM-2"[Title/Abstract]) AND ("Mitral Valve"[Mesh] OR "Mitral Valve" OR "Mitral Valve Insufficiency"[Mesh] OR "Mitral Valve Insufficiency" OR "Mitral Regurgitation" OR "Mitral Valve Repair" OR "Transcatheter Edge-to-Edge Repair" OR "TEER" OR "MitraClip")) | 83 |
| Embase | (("Diabetes"[Mesh] OR "Diabetes type-2" OR "DM"[Title/Abstract] OR "DM-  2"[Title/Abstract])  AND  (mitral valve/exp OR 'mitral valve' OR 'mitral valve insufficiency/exp OR 'mitral valve  insufficiency' OR 'mitral regurgitation' OR 'mitral valve repair' OR 'transcatheter edge-to-edge  repair' OR 'teer' OR 'mitraclip')  AND  ('outcome' OR 'outcomes' OR 'prognosis/exp OR 'prognosis' OR 'short-term' OR 'long-term' OR "follow up/exp OR 'follow-up') | 96 |

**Supplemental S4: Risk of bias (ROB) assessment for randomized control trials**

**
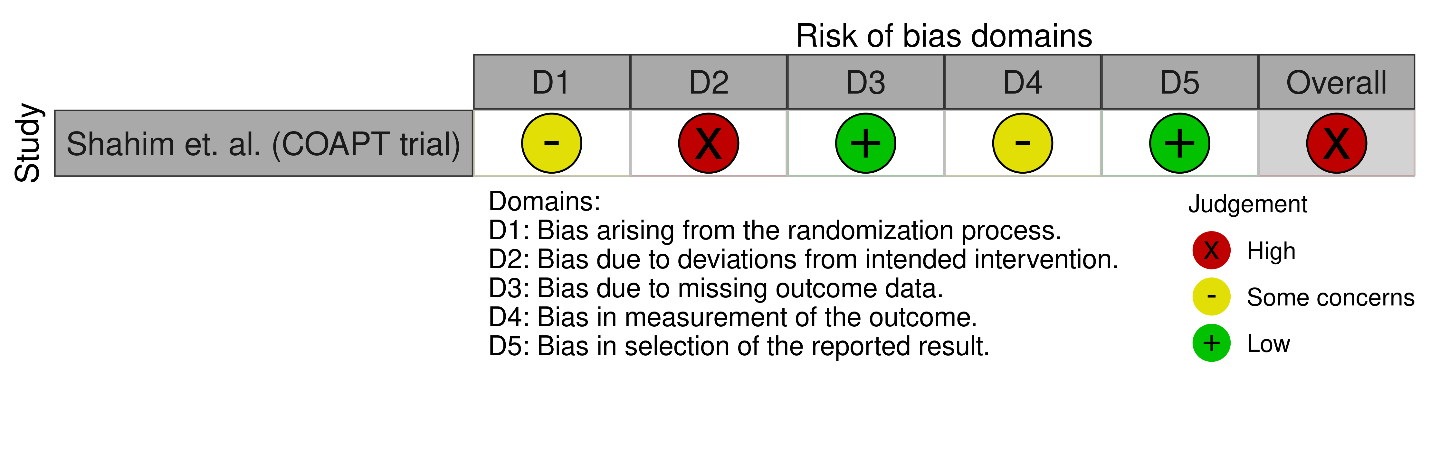
**

**Supplemental S5: Newcastle-Ottawa Scale for assessment of bias for observational studies**

| Study | Representativeness of the exposed cohort | Selection of the non-exposed cohort | Ascertainment of exposure | Demonstration that outcome of interest was not present at start of study | Comparability of cohorts on the basis of the design or analysis | Assessment of outcome | Adequate follow-up | Adequacy of follow-up cohort | Total |
| --- | --- | --- | --- | --- | --- | --- | --- | --- | --- |
| Kirschfink et. al. | 1 | 1 | 1 | 1 | 2 | 1 | 0 | 1 | 8 |
| Hellhammer et. al. | 1 | 1 | 1 | 1 | 1 | 1 | 0 | 1 | 7 |
| Paukovitsch et. al. | 1 | 1 | 1 | 1 | 2 | 1 | 0 | 1 | 8 |

**Supplemental S6: Egger’s test and funnel plots to assess publication bias**

- **Egger’s test and Funnel plot for 30-day all-cause mortality**

**
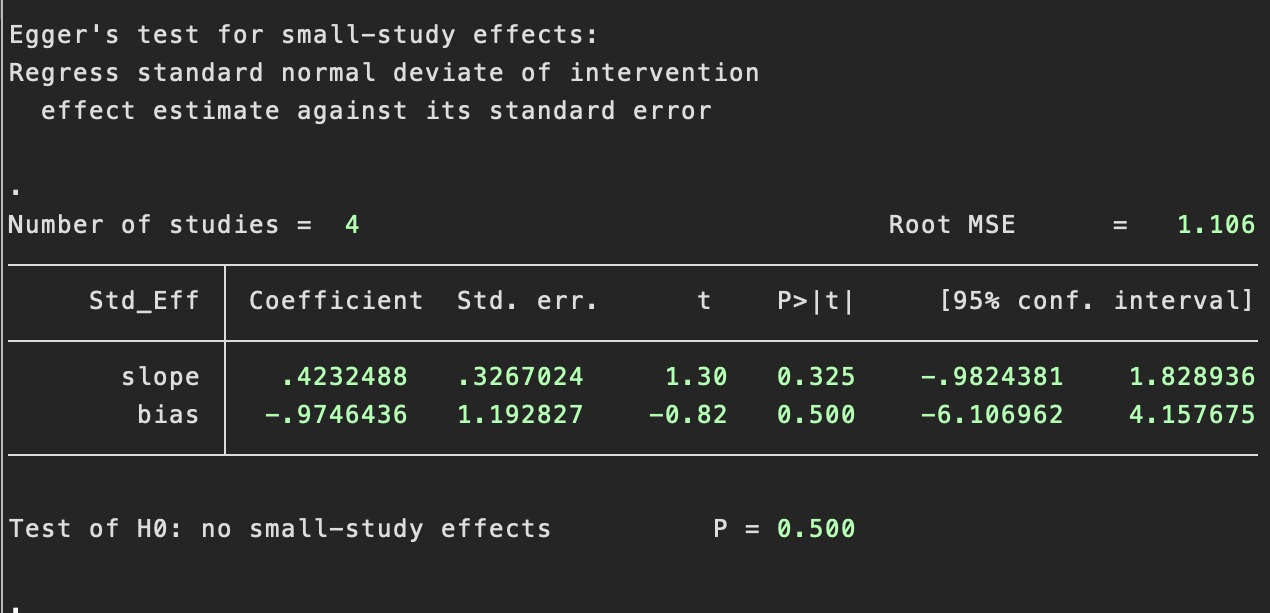
**

**
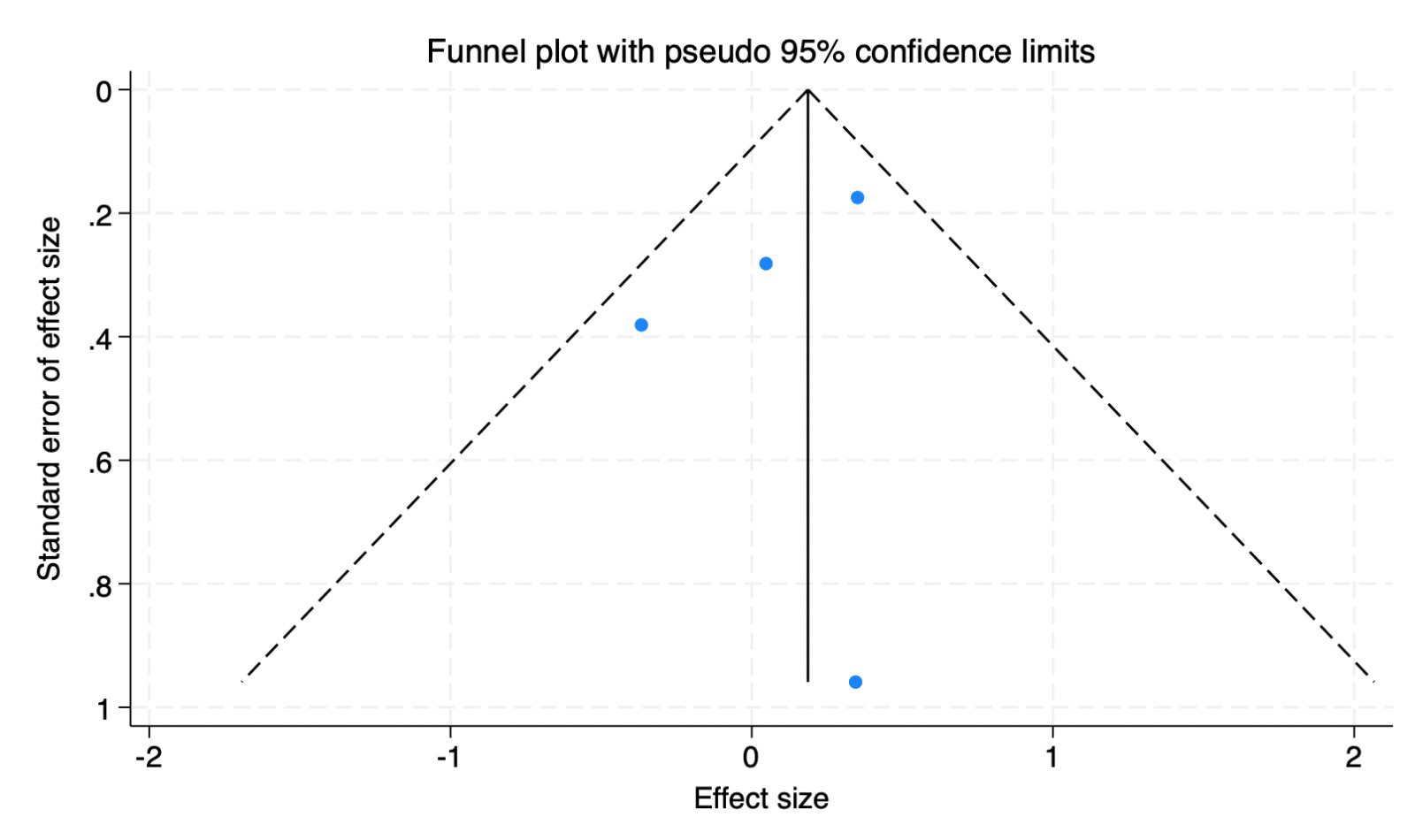
**

- **Egger’s test and Funnel plot for 30-day MACCE**


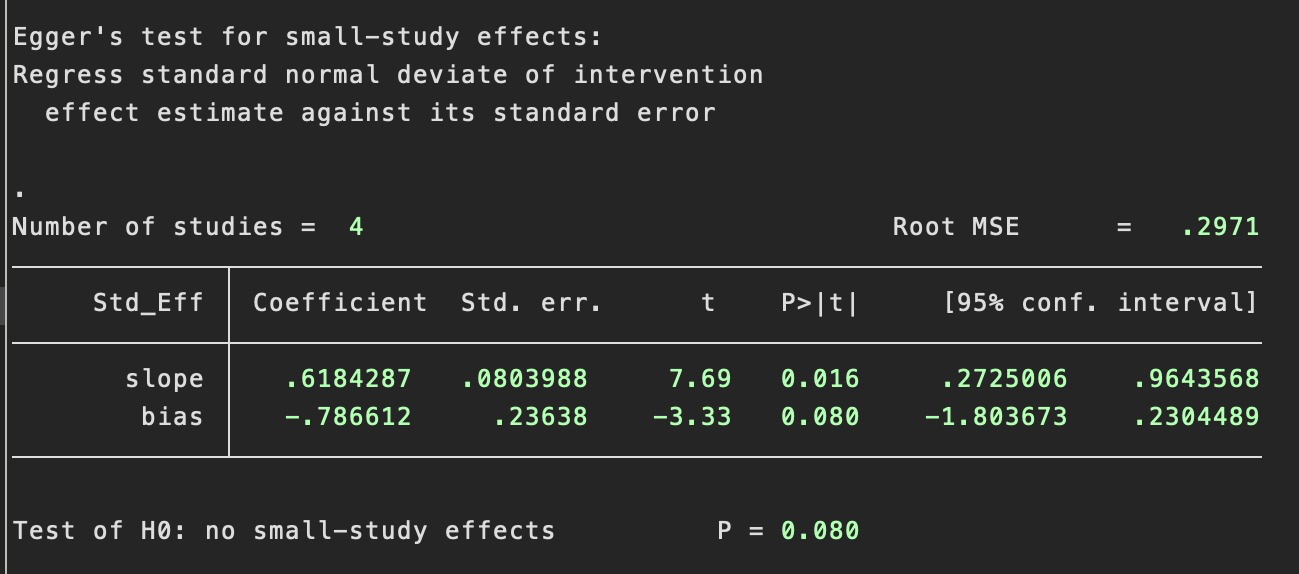


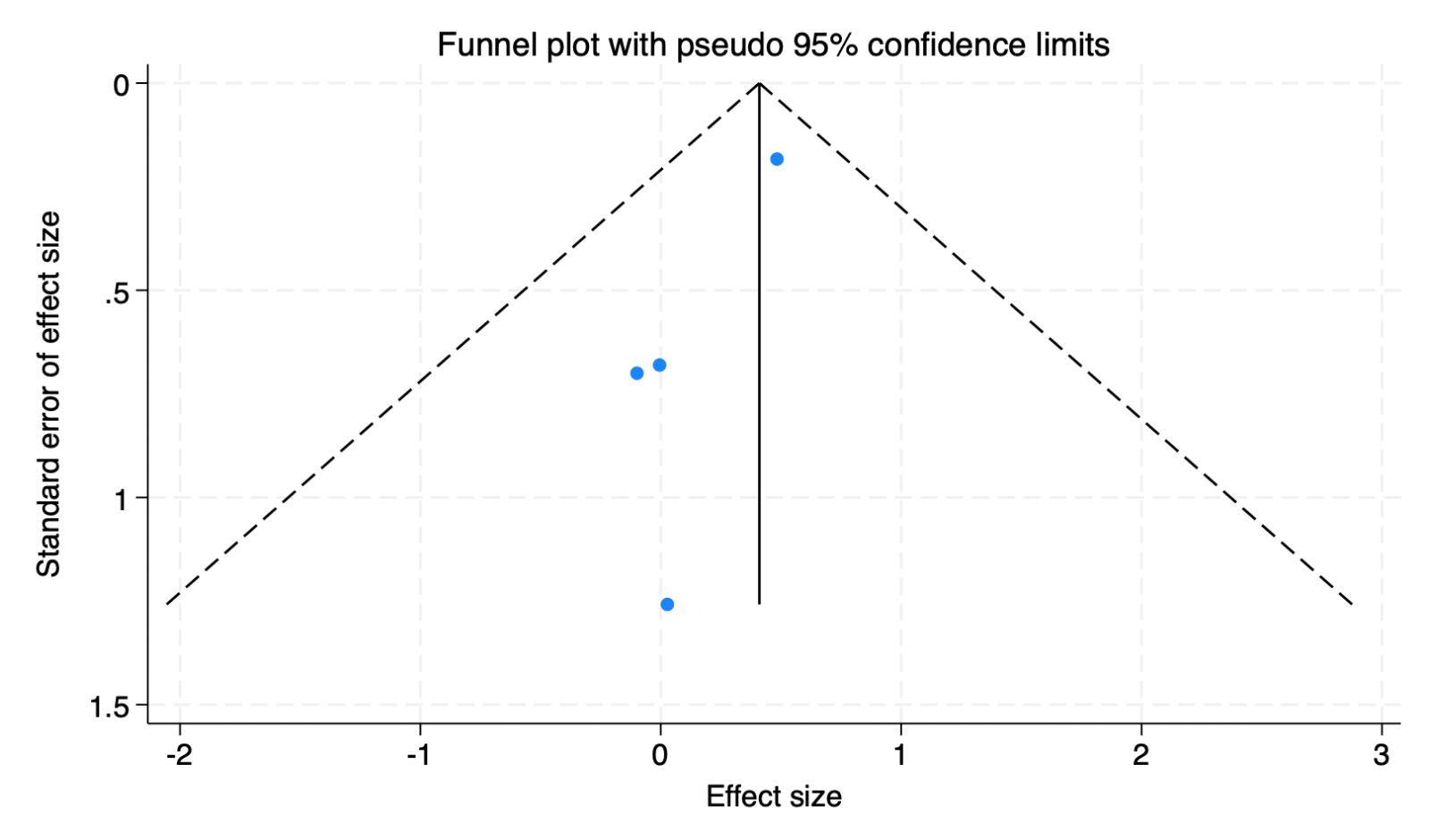


- **Egger’s test and Funnel plot for In-hospital mortality**

**
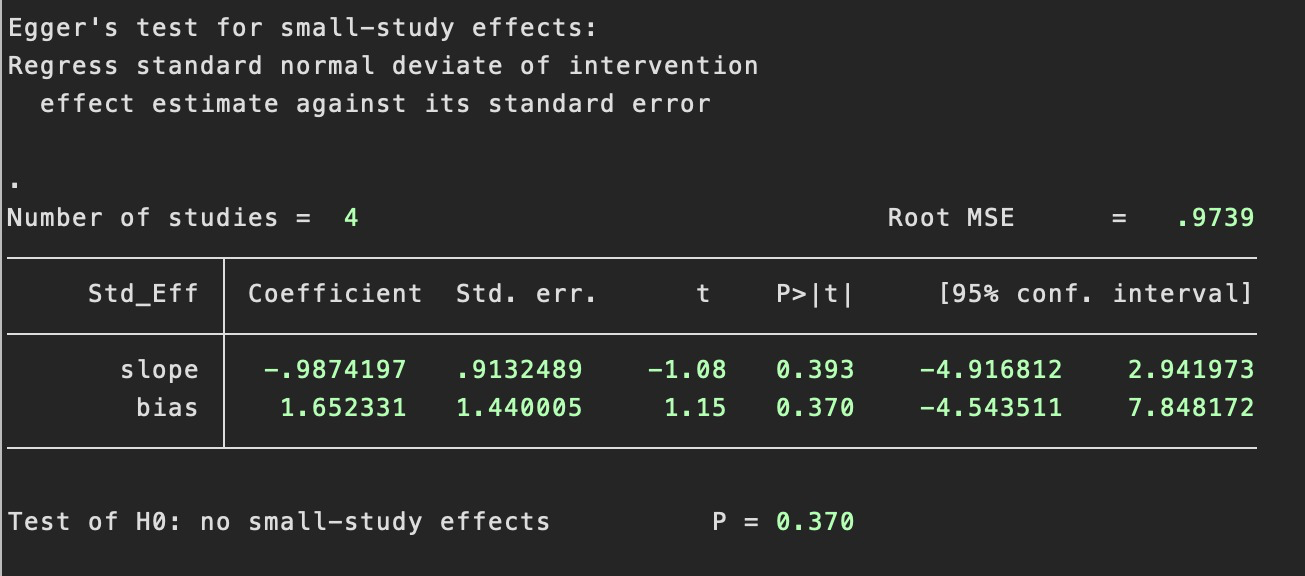
**

**
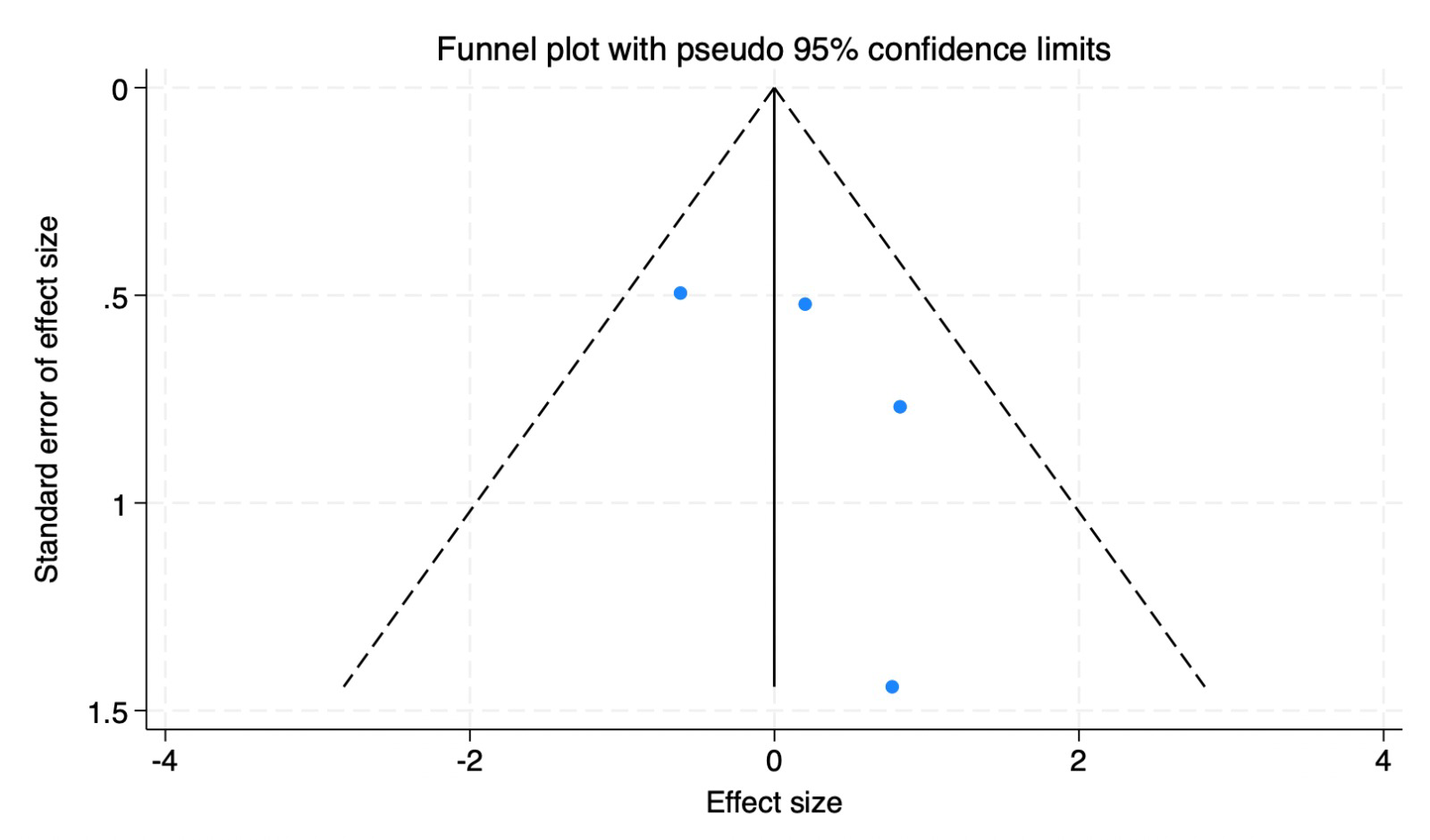
**

- **Egger’s test and Funnel plot for 30-day recurrent hospitalization**

**
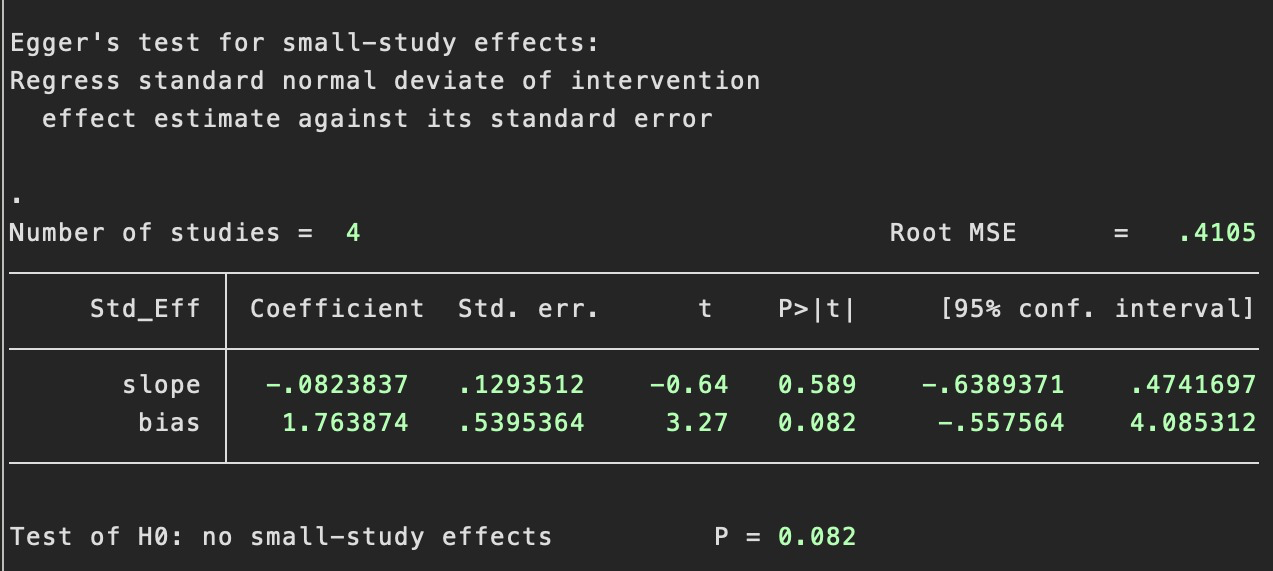
**

**
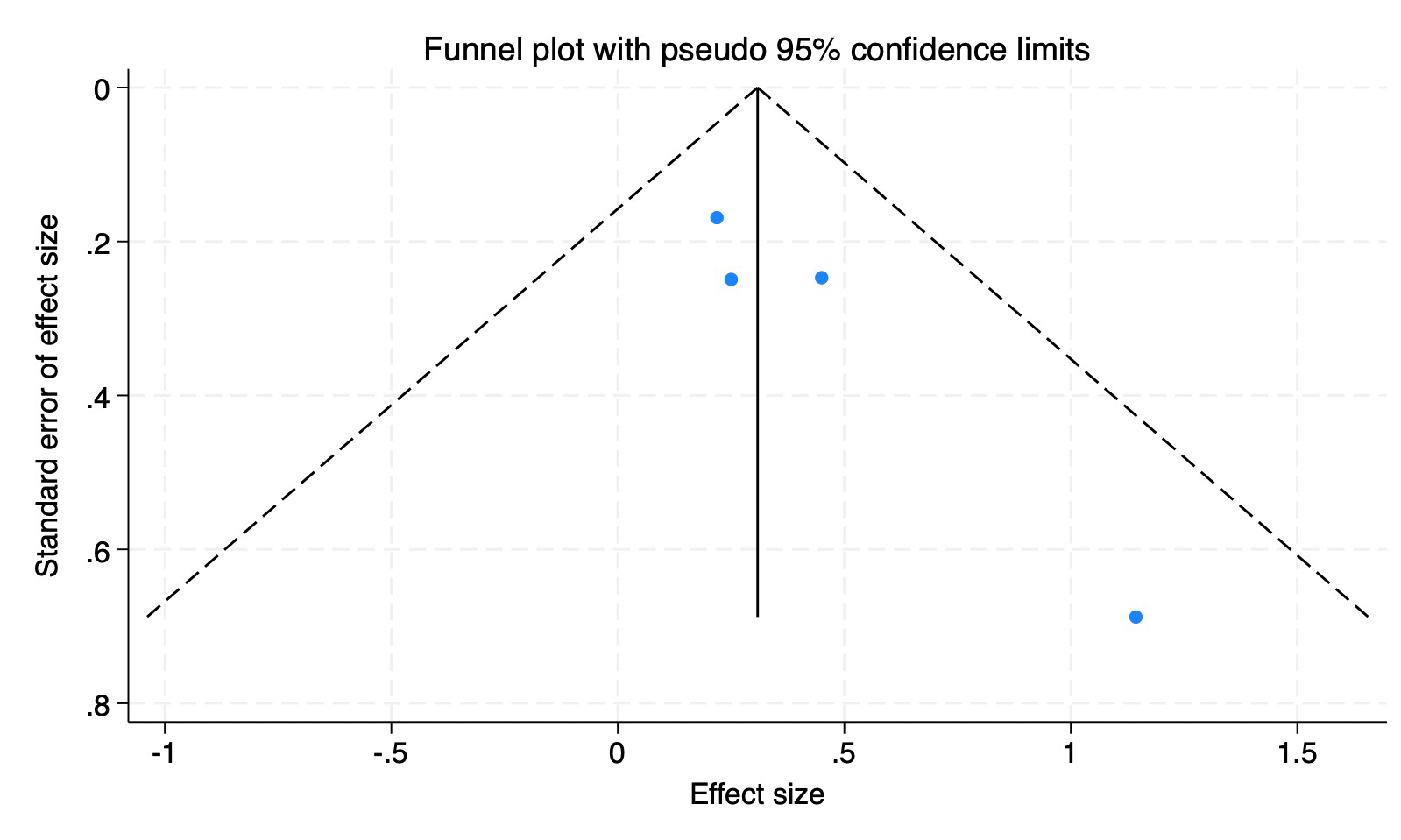
**

**Supplemental S7: Leave one-out Sensitivity**

**30-day all-cause mortality**

**30-day MACCE**

**30-day all-cause recurrent hospitalizations**

**Supplemental S8: Table for the definitions:**

| ***Variable*** | ***Definition*** |
| --- | --- |
| *All-Cause Mortality* | *Death from any cause* |
| *MACCE* | *Major Adverse Cardiac and Cerebrovascular Events (Includes Cardiovascular death, Acute Myocardial Infarction and cardiac revascularization procedures).* |
| *HF hospitalizations* | *Patients admitted with heart failure following the procedure* |
| *LOS* | *The entire length of stay the patient spent in the hospital during the admission* |
| *Cost of hospitalization* | *The total amount that hospitals billed for their services to the patient for the duration of hospitalization* |
| *MR* | *Mitral Regurgitation* |
| *MR >1* | *refers to post-procedural MR reduction greater than one grade, indicating significant improvement.* |
